# Three-years outcomes of diabetic patients treated with coronary bioresorbable scaffolds

**DOI:** 10.1186/s12872-018-0811-7

**Published:** 2018-05-10

**Authors:** Remzi Anadol, Katharina Schnitzler, Liv Lorenz, Melissa Weissner, Helen Ullrich, Alberto Polimeni, Thomas Münzel, Tommaso Gori

**Affiliations:** Kardiologie I, Zentrum für Kardiologie, German Center for Cardiac and Vascular Research (DZHK), Standort Rhein-Main, University Hospital Mainz, Langenbeckstraße 1, 55131 Mainz, Germany

**Keywords:** Diabetes, Bioresorbable scaffolds, Coronary artery disease

## Abstract

**Background:**

Diabetes is among the strongest predictors of outcome after coronary artery stenting and the incidence of negative outcomes is still high in this specific group. Data of long-term outcomes comparing diabetic patients with non-diabetic patients treated with bioresorbable scaffolds are still incomplete. This work evaluates the long-term outcomes after implantation of a coronary bioresorbable scaffold (BRS) in diabetic patients compared to non-diabetics.

**Methods:**

Patients who received at least one Absorb BRS in the time of May 2012 to December 2014 were enrolled into this single-center registry. Quantitative coronary angiography (QCA) was performed.

**Results:**

Six hundred fifty seven patients including 138 patients (21%, mean age 65 ± 11, 78% male) with diabetes were enrolled.

Patients in the diabetic group were significantly older, were more likely to suffer from hypertension and hyperlipidemia and had more often a prior stroke or TIA as well as a reduced renal function (all *P* < 0.05). The initial stenosis was less severe in the diabetic group (74.8% vs. 79.6%, *P* = 0.036), but the residual stenosis after BRS implantation exceeded that of the control group (16.7% vs. 13.8%, *P* = 0.006).

History of diabetes had no impact on the incidence of events within one year after BRS implantation. Beyond 1 year, diabetic patients had a higher incidence of cardiovascular death (6.9 vs. 1.4%, HR:5.37 [1.33–21.71], *P* = 0.001), scaffold restenosis (17.6 vs. 7.8%, HR:3.56 [1.40–9.05], *P* < 0.0001) and target lesion revascularization (*P* = 0.016). These results were confirmed in the propensity score analysis.

In both diabetics and non-diabetics, there was a strong association (HR:18.6 [4.7–73.3]) between the risk of restenosis and the technique used at implantation; in contrast, the impact of vessel size was more manifest in non-diabetics than in diabetic patients, and an increased risk of restenosis was demonstrated for both large and small vessels.

**Conclusion:**

As for metal stents, beyond one year after implantation, diabetes was associated with an increased incidence of scaffold restenosis and related outcomes. This negative impact of diabetes was reset when an optimal implantation technique was used.

**Electronic supplementary material:**

The online version of this article (10.1186/s12872-018-0811-7) contains supplementary material, which is available to authorized users.

## Background

Due to the higher prevalence of complex clinical and angiographic features, diabetes is among the strongest predictors of outcome after coronary artery stenting [1]. While being associated with a significant improvement as compared to bare metal stents, the rate of target-lesion failures following use of modern drug eluting stents in diabetics still remains as high as 2–4% per year, and diabetes remains an important factor in the decision regarding the revascularization strategy to be followed, particularly in patients with multivessel disease [2, 3].

Bioresorbable coronary scaffolds (BRS) were developed to reduce late-occurring complications of stenting and were brought to the EU market in 2012 without restriction for the type of (de novo) lesion or the clinical setting in which they were to be used [4]. Particularly in the setting of diabetes, where the chronic inflammatory environment is felt to be associated with stent failure [5], the resorption of the scaffold strut might theoretically provide an advantage over permanent metallic devices. Recent data [6–8], however, point out at increased rates of target lesion failure in patients treated with BRS as compared to those treated with newer generation drug eluting stents, an observation which lead first to the restriction of BRS to registries and then to the removal of Absorb BRSs from the market. While evidence regarding the role of procedural parameters has been well investigated [9, 10], less evidence is available regarding clinical features as predictors of risk after BRS implantation. In particular, diabetes has been shown to be a predictor of target lesion failure in registry studies with a 6-months to 1-year follow-up [11–13], but not in a pooled analysis of the ABSORB cohort B, ABSORB Extend and SPIRIT trials [14]. In a smaller study with a 3-years follow-up [15], numerically more target lesion failures were reported, but the difference against non-diabetic patients was not significant. Other than this study, data on long-term outcomes are missing. Such data are particularly important because the risk of restenosis, and therefore the impact of diabetes, would be expected to increase later than one year after implantation. In the present paper, we report on the 3- to 4-years outcomes of patients with diabetes treated with Absorb BRS in a larger cohort of consecutive patients.

## Methods

### Study design

This investigator-initiated, single-centre, single-arm observational study enrolled consecutive patients who received one or more Absorb BRS (Abbott Vascular, Santa Clara, CA, USA) at the University hospital of Mainz between May 2012 and December 2014. The outcome after BRS implantation in patients with diabetes mellitus was compared to that of non-diabetes patients.

Follow-up data were obtained in the setting of office visits or telephone calls and entered retrospectively in the research database. Trained medical staff obtained data using a standardized questionnaire. In the case of hospital admission or events, original medical documents were obtained from the patients, treating cardiologists or general practitioners. Events were adjudicated by consensus of at least two experienced interventionalists. The study belongs to the MICAT project (NCT02180178), which is approved by the local ethics committee.

### BRS implantation

As published before [16], BRS were not used for stenoses in the left main coronary or vein grafts, in-stent restenosis, bifurcation lesions with side branches > 2 mm. Furthermore, patients with intolerance to aspirin or thienopyridines, pre-existing therapy with anticoagulants or patients with limited life expectancy received no BRS. Predilatation was systematically used; postdilatation was systematically used starting from January 2014. Dual antiplatelet therapy for 12 months (aspirin plus clopidogrel for stable disease; aspirin plus prasugrel/ticagrelor for acute coronary syndromes) was recommended.

### Quantitative coronary analysis

Quantitative coronary analysis (QCA) was performed off-line by trained personnel blinded to the patients´ clinical characteristics and outcomes. Parameters included lesion length, interpolated reference vessel diameter (RVD) and minimum lumen diameter (MLD) before and after implantation. Initial stenosis, residual stenosis and lumen gain was calculated by these parameters:

$$ \mathrm{Initial}/\mathrm{residual}\ \mathrm{stenosis}=1\hbox{--} \mathrm{MLD}/\mathrm{RVD} $$

$$ \mathrm{Lumen}\ \mathrm{gain}=\mathrm{MLD}\ \mathrm{postprocedural}\hbox{--} \mathrm{MLD}\ \mathrm{preprocedural} $$

Xcelera R 4.1 (Philips, the Netherlands) was used for these measurements; detailed methods, reproducibility and repeatability data have already been published before [17].

### Endpoints

Events were defined according to the academic research consortium definitions [18]. Events were analysed separately for early (< 30 days), late (31–365 days), very late (> 365 days) timepoints and as overall occurrence. Endpoints included death, cardiovascular death (CV death), myocardial infarction (MI) and target vessel MI (TVMI), target lesion revascularization (TLR), target vessel revascularization (TVR), clinically relevant scaffold restenosis (ScR) and scaffold thrombosis (ScT). TLR was defined as any revascularization of the original segment (scaffold + 5 mm proximal and distal). CV death, TVMI and TLR were analyzed together as device-oriented composite endpoint (DoCE).

### Statistical methods

Continuous data were presented as mean and standard deviation (SD) or median (interquartile range) and are analysed with parametric or non-parametric tests as appropriate; categorical data are presented as total numbers and proportions and were analysed with Chi square or Fisher’s exact test. Analysis of the outcome of diabetes patients compared to non-diabetes patients treated with BRS was performed with cox regression analysis and is illustrated in Kaplan-Meier curves. All variables listed in Table 1 were tested at univariate level and those with a *P* < 0.1 were introduced in a multivariable model. To remove potential bias, propensity score analysis was performed for DoCE, ScR and ScT using the inverse probability treatment weighted analysis method and average treatment effect adjustment. This method uses propensity score-based weights to create a sample in which the distribution of covariates is independent of the group. Parameters for the propensity score weights included gender, hypertension, smoking, family history, hyperlipidemia, prior CABG, prior PCI, prior stroke or transient ischemic attack, estimated glomerular filtration rate.

**Table 1:** Patient and procedural characteristics

	All patients (*n* = 657)	Patients with diabetes (*n* = 138)	Patients without diabetes (*n* = 519)	*P*-value
Age (years)	63 ± 12	65 ± 11	62 ± 12	**0.015**
Male	519/657 (79.0%)	107/138 (77.5%)	412/519 (79.4%)	0.722
Hypertension	478/657 (72.8%)	117/138 (84.8%)	361/519 (69.6%)	**< 0.001**
Smoking	273/657 (41.6%)	48/138 (34.8%)	225/519 (43.3%)	0.086
Family History	138/657 (21.0%)	21/138 (15.2%)	117/519 (22.5%)	0.078
Hyperlipidemia	268/657 (40.8%)	76/138 (55.1%)	192/519 (37.0%)	**< 0.001**
Prior CABG	14/657 (2.1%)	5/138 (3.6%)	9/519 (1.7%)	0.186
Prior PCI	181/657 (27.5%)	48/138 (34.8%)	133/519 (25.6%)	**0.042**
Prior stroke/TIA	27/657 (4.1%)	11/138 (8.0%)	16/519 (3.1%)	**0.020**
eGFR (ml/min)	83 ± 23	76 ± 25	84 ± 22	**< 0.001**
LVEF (%)	52 ± 8	53 ± 7	52 ± 9	0.590
Silent/stable angina	219/657 (33.3%)	56/138 (40.6%)	163/519 (31.4%)	0.054
Unstable Angina	78/657 (11.9%)	20/138 (14.5%)	58/519 (11.1%)	0.356
NSTEMI	191/657 (29.1%)	33/138 (23.9%)	158/519 (30.4%)	0.163
STEMI	166/657 (25.3%)	28/138 (20.3%)	138/519 (26.6%)	0.161
Lesion characteristics				
LAD treated with BRS	301/657 (45.8%)	68/138 (49.3%)	233/519 (44.9%)	0.411
LCX treated with BRS	161/657 (24.4%)	36/138(26.1%)	125/519(24.1%)	0.708
RCA treated with BRS	194/657 (29.7%)	34/138 (24.6%)	160/519 (30.8%)	0.190
Graft treated with BRS	1/657 (0.2%)	0/138 (0%)	1/519 (0.2%)	1
Ostial lesion	53/657 (8.1%)	10/138 (7.2%)	43/519 (8.3%)	0.824
CTO	11/657(1.7%)	2/138 (1.4%)	15/519 (2.9%)	0.546
Bifurcation	82/657(12.5%)	13/138(9.4%)	69/519(13.3%)	0.281
At least one lesion type B2 or C	297/657 (45.2%)	62/138 (44.9%)	235/519 (45.3%)	0.982
Procedural characteristics				
Number of vessels treated with BVS	1.2 ± 0.4	1.2 ± 0.5	1.1 ± 0.4	0.095
Number of BRS per patient	1.4 ± 0.8	1.5 ± 0.9	1.4 ± 0.8	0.181
vHybrid BRS + DES	330/657(50.2%)	56/138(40.6%)	274/519(52.8%)	**0.014**
Predilatation	653/657(99.4%)	136/138(98.6%)	517/519(99.6%)	0.196
Diameter predilatation ballon (mm)	2.80 ± 0.37	2.83 ± 0.36	2.80 ± 0.37	0.367
Minimum stent diameter per patient (mm)	2.97 ± 0.38	2.96 ± 0.36	2.98 ± 0.38	0.572
Total implanted length per patient (mm)	27.5 ± 18.9	29.0 ± 19.4	27.2 ± 18.8	0.324
Postdilatation	306/657(46.6%)	71/138(51.4%)	235/519(45.3%)	0.232
Preprocedural RVD, mm	2.93 ± 0.67	2.91 ± 0.77	2.94 ± 0.65	0.728
Preprocedural MLD, mm	0.61 ± 0.51	0.71 ± 0.41	0.58 ± 0.54	**0.026**
% stenosis per lesion	78.2 ± 17.9	74.6 ± 13.6	79.4 ± 18.8	**0.014**
Angiographic Outcome				
Postprocedural RVD, mm	3.0 ± 0.5	3.0 ± 0.4	3.0 ± 0.5	0.760
vPostprocedural MLD, mm	2.5 ± 0.5	2.5 ± 0.5	2.5 ± 0.5	0.135
Residual stenosis per lesion (%)	14.5 ± 10.8	16.7 ± 13.2	13.7 ± 9.9	**0.018**
MLD/nominal BRS diameter	0.84 ± 0.13	0.82 ± 0.16	0.85 ± 0.13	0.101
Lumen Gain, mm	1.63 ± 0.62	1.61 ± 0.61	1.68 ± 0.56	0.382
Optimal implantation technique	311/657 (47.3%)	74/138 (53.6%)	237/519 (45.7%)	0.117
Overlap	74/657 (11.3%)	12/138 (8.7%)	62/519 (11.9%)	0.357
Clopidogrel	200/657 (30.4%)	51/138 (37.0%)	149/519 (28.7%)	0.077
Prasugrel	324/657 (49.3%)	67/138 (48.6%)	257/519 (49.5%)	0.915
Ticagrelor	132/657 (20.1%)	19/138 (13.8%)	113/519 (21.8%)	0.076

*P* < 0.05 as statistically significant are in bold

In order to address the role of the implantation technique used, the following procedural predictors were investigated separately and as a whole:

1. Predilatation with a balloon of the same nominal size as the BRS.
2. Vessel size (RVD) comprised between 2.5–3.5 mm
3. BRS sizing: implantation of a BRS of the same size as the reference vessel diameter (nominal diameter to reference vessel diameter ratio comprised between 0.9 and 1.1).
4. Postdilatation at 14-16ATM with noncompliant balloons of the same size or 0.5 mm larger than the BRS.

Previous studies have shown that this technique is associated with a lower incidence of both ScT and ScR [10, 19].

The significance level was set at *p* < 0.05. MedCalc Version 9.2.1.0 (Mariakerke, Belgium) and R Statistical Software (Foundation for Statistical Computing, Vienna, Austria) were used for the analysis.

## Results

### Patients and baseline characteristics

Clinical and procedural characteristics are shown in Table 1. Of a total of 657 patients, 138 (21%) patients were diabetics and 519 (79%) were non-diabetics. Patients in the diabetic group were older (*p* = 0.016), had a higher prevalence of hypertension (85% vs. 70%, *p* = 0.0005), hyperlipidemia (55% vs. 37%, *p* = 0.0002) and prior stroke or TIA (8% vs. 3%, *p* = 0.0198). Furthermore, the estimated glomerular filtration rate was lower in the diabetic group (76 ± 25 ml/min vs. 84 ± 22, *p* < 0.001).

### QCA and angiographic outcome

Although the initial stenosis tended to be less severe in the diabetic group (diabetic 74.8 ± 13.6% vs control 79.6 ± 18.5%, *p* = 0.036), the residual stenosis after BRS implantation in the diabetic group exceed that in the control group (16.7 ± 13.3% vs. 13.8 ± 9.9%, *p* = 0.006). Correspondingly, the lumen gain was smaller in the diabetic group (1.92 ± 0.9 vs. 2.17 ± 0.7 mm, *p* = 0.001). As a parameter of BRS expansion and marker of prognosis [16], the quotient of MLD to the nominal BRS diameter was not different between groups.

### Follow-up

Data are presented in Table 2. The median[IQR] follow-up was 1044 [763–1198] days in diabetics and 1084 [762–1207] days in non-diabetics (*P* = 0.328). Although numerically higher in the diabetic group, the incidence of events was not significantly different between diabetic and non-diabetic patients at 30 days and within the first year after BRS implantation. After one year, the incidence of CV death, ScR and TLR were higher in the diabetic group (all *P* < 0.05). Diabetes did not impact overall mortality, myocardial infarction and scaffold thrombosis. In multivariable analysis, diabetes (HR 2.89 [1.55–5.39], *P* = 0.0009) was an independent predictor of ScR along with prior revascularization, type B2/C lesions and the technique used at implantation. The propensity score (Table 3) at both the lesion and patient level confirmed the independent association between DoCE and ScR rates in diabetics, while the incidence of ScT was not different between groups. Patient and lesion characteristics after propensity score adjustment are presented in Additional file 1: Table S1a and b.

**Table 2:** Patient outcomes

	Within 30 Days					Between 31 Days and 1 year					After 1 year				
	All	Diab	No Diab	P	HR [95% CI]	All	Diab	No Diab	P	HR [95% CI]	All	Diab	No Diab	p	HR [95% CI]
All Death	12 (1.9%)	2 (1.5%)	10 (2.0%)	0.713	0.75 [0.19–3.02]	7 (1.2%)	1 (0.8%)	6 (1.3%)	0.666	0.63 [0.10–3.89]	24 (5.2%)	8 (7.7%)	16 (4.5%)	0.129	1.91 [0.71–5.11]
CV Death	7 (1.1%)	2 (1.5%)	5 (1.0%)	0.623	1.50 [0.24–9.26]	5 (0.8%)	0	5 (1.0%)	0.250	–	**12 (2.5%)**	**7 (6.9%)**	**5 (1.4%)**	**0.001**	**5.37 [1.33–21.71]**
Any MI	12 (1.9%)	4 (3.0%)	8 (1.6%)	0.286	1.90 [0.47–7.64]	16 (2.7%)	4 (3.3%)	12 (2.6%)	0.661	1.29 [0.38–4.32]	25 (5.5%)	6 (6.4%)	19 (5.3%)	0.705	1.19 [0.46–3.13]
Target Vessel MI	10 (1.6%)	3 (2.2%)	7 (1.4%)	0.477	1.62 [0.35–7.46]	12 (2.0%)	4 (3.3%)	8 (1.7%)	0.275	1.93 [0.48–7.81]	15 (2.8%)	3 (2.6%)	12 (2.8%)	0.928	0.94 [0.27–3.27]
ScR	–	–	–	–	–	13 (2.2%)	5 (4.0%)	8 (1.7%)	0.116	2.38 [0.62–9.09]	**28 (10.3%)**	**13 (17.6%)**	**15 (7.8%)**	**< 0.001**	**3.56 [1.40–9.05]**
ScT	14 (2.2%)	3 (2.2%)	11 (2.2%)	0.963	1.03 [0.28–3.73]	5 (0.9%)	0	5 (1.1%)	0.251	–	9 (1.7%)	1 (1.4%)	8 (1.8%)	0.459	0.46 [0.09–2.30]
TLR	–	–	–	–	–	22 (3.7%)	6 (4.9%)	16 (3.4%)	0.443	1.44 [0.51–4.04]	**33 (11.5%)**	**12 (17.0%)**	**21 (9.5%)**	**0.016**	**2.32 [0.98–5.47]**

*P* < 0.05 as statistically significant are in bold

**Table 3:** Propensity score analysis

	Patient level				Lesion level			
	Unadjusted		Average treatment effect-adjusted		Unadjusted		Average treatment effect -adjusted	
	p	HR 95% CI	p	HR 95% CI	p	HR 95% CI	p	HR 95% CI
DoCE	0.026	1.74 [1.07–2.83]	0.0026	1.59 [1.18–2.16]	0.045	1.65 [1.01–2.68]	0.003	1.58 [1.17–2.13]
ScT	0.6	0.75 [0.26–2.18]	0.43	0.79 [0.44–1.42]	0.52	0.7 [0.24–2.05]	0.2	0.68 [0.37–1.23]
ScR	0.0067	2.52 [1.29–4.91]	0.00038	2.2 [1.42–3.41]	0.0051	2.37 [1.3–4.35]	0.00077	1.97 [1.33–2.92]

### Implantation technique

**Fig. 1:**
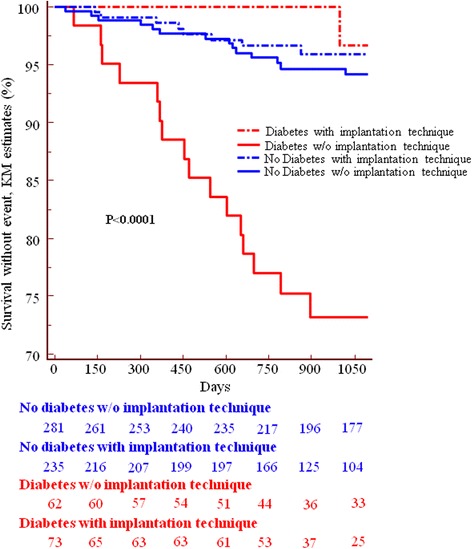
Incidence of scaffold restenosis in patients with diabetes and patients without diabetes and effect of the implantation technique. The incidence of ScR was higher in diabetics, but the application of a “full” implantation technique reduced it in diabetics but not in non-diabetics

**Fig. 2:**
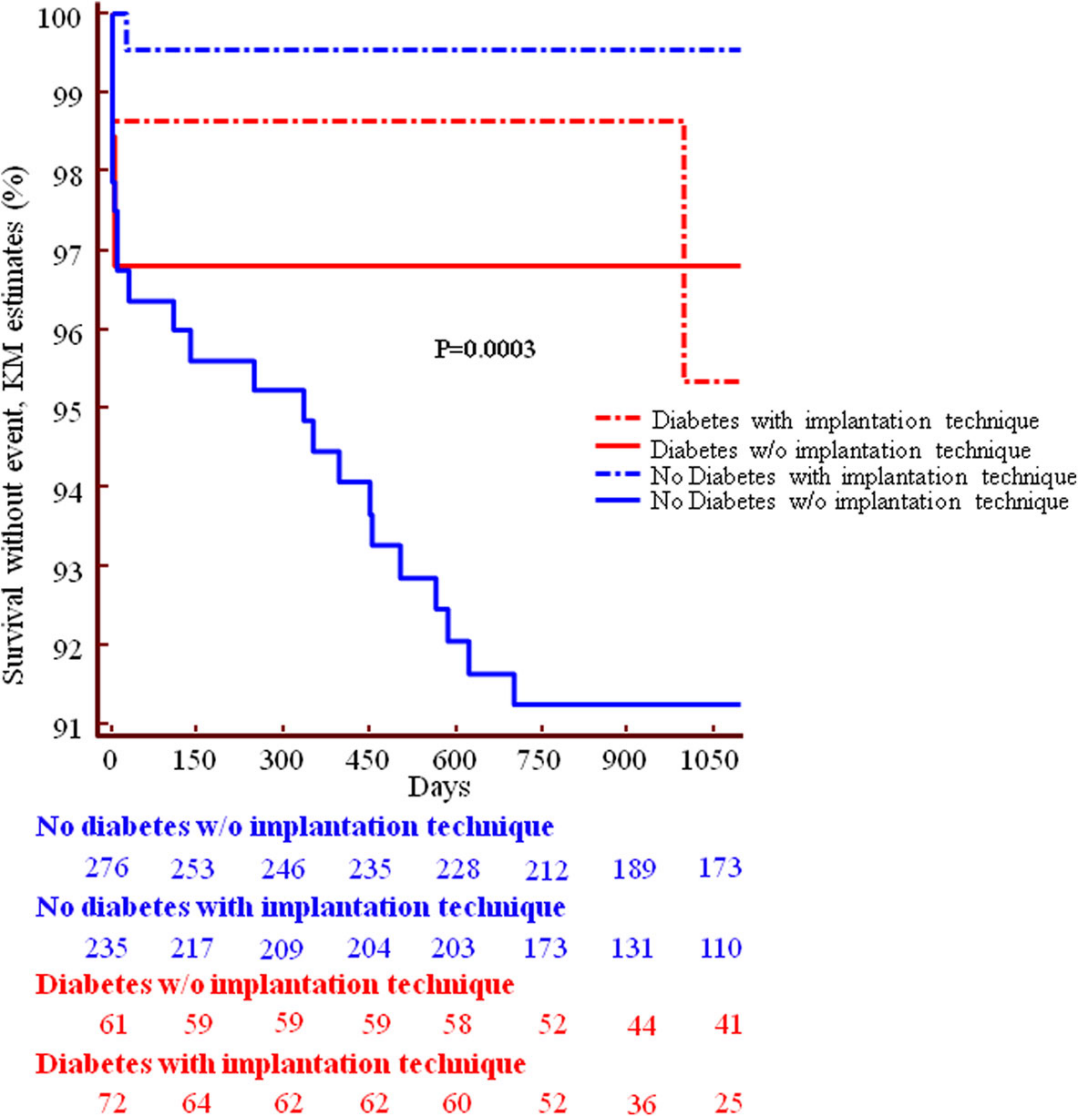
Incidence of scaffold thrombosis in patients with diabetes and patients without diabetes and effect of the implantation technique. The incidence of ScT was not significantly different in diabetics and non-diabetics, although a trend towards a paradoxically lower incidence of late ScT in diabetics was shown. The implantation technique reduced the incidence of ScT in non-diabetics more than in diabetics

The full implantation technique as described above was used in 74 patients (54%) of the diabetic and in 237 patients of the control group (46%) (*p* = 0.117 between groups). Outcome data are presented in Figs. 1, 2, and Tables 4 and 5. In diabetic patients, application of an optimal implantation technique was associated with reduced incidence of ScR, ScT, TV-MI, TLR and TVR. Table 5 presents the separated HRs for the impact of the implantation technique in the two patient groups. Interestingly, a suboptimal implantation technique was associated with a marked increase in the risk of ScR (HR 18.6[4.7–73.3], from 27% to 1%) but not of ScT (3% for both) in diabetic patients. In contrast, the implantation technique had a marked impact on ScT (but not ScR) in non-diabetic patients (Table 5).

**Table 4:** Impact of the implantation technique in patients with diabetes

Diabetes	Implantation Technique			
	No (63)	Yes (74)	p	HR 95% KI
all death	4/63 6%)	7/74 (9%)	0.3415	0.56 0.17–1.84
CV death	3/63 (5%)	6/74 (8%)	0.284	0.48 0.13–1.81
any MI	10/63 (16%)	5/74 (7%)	0.1207	2.28 0.81–6.17
TV- MI	9/63 (14%)	1/74 (1%)	**0.006**	10.27 1.64–19.80
TLR	19/63 (30%)	2/74 (3%)	**0.0001**	10.79 22.49–13.97
TVR	25/63 (40%)	7/74 (9%)	**0.0001**	4.41 1.94–7.86
ScR	17/63 (27%)	1/74 (1%)	**0.0001**	18.54 2.61–16.80
ScT	2/63 (3%)	2/74 (3%)	0.9722	1.03 0.14–7.45
DOCE	22/63 (35%)	8/74 (11%)	**0.0021**	3.29 1.51–6.41

*P* < 0.05 as statistically significant are in bold

**Table 5:** Hazard Ratios with respective 95% confidence interval of the effect of the implantation technique in the two groups

	Diabetes without vs. Diabetes with optimal implantation	Diabetes vs No diabetes, both with optimal implantation	Diabetes vs No Diabetes, both without optimal implantation	No diabetes without vs. no diabetes with optimal implantation
ScT	1.083 [0.215–5.458]	**6.641 [1.849–23.854]**	0.369 [0.101–1.348]	**19.503 [8.451–45.010]**
ScR	**18.596 [4.719–73.284]**	0.419 [0.142–1.233]	**5.360 [1.800–15.956]**	1.452 [0.728–2.895]

*P* < 0.05 as statistically significant are in bold

### Relationship with the vessel diameter

**Fig. 3:**
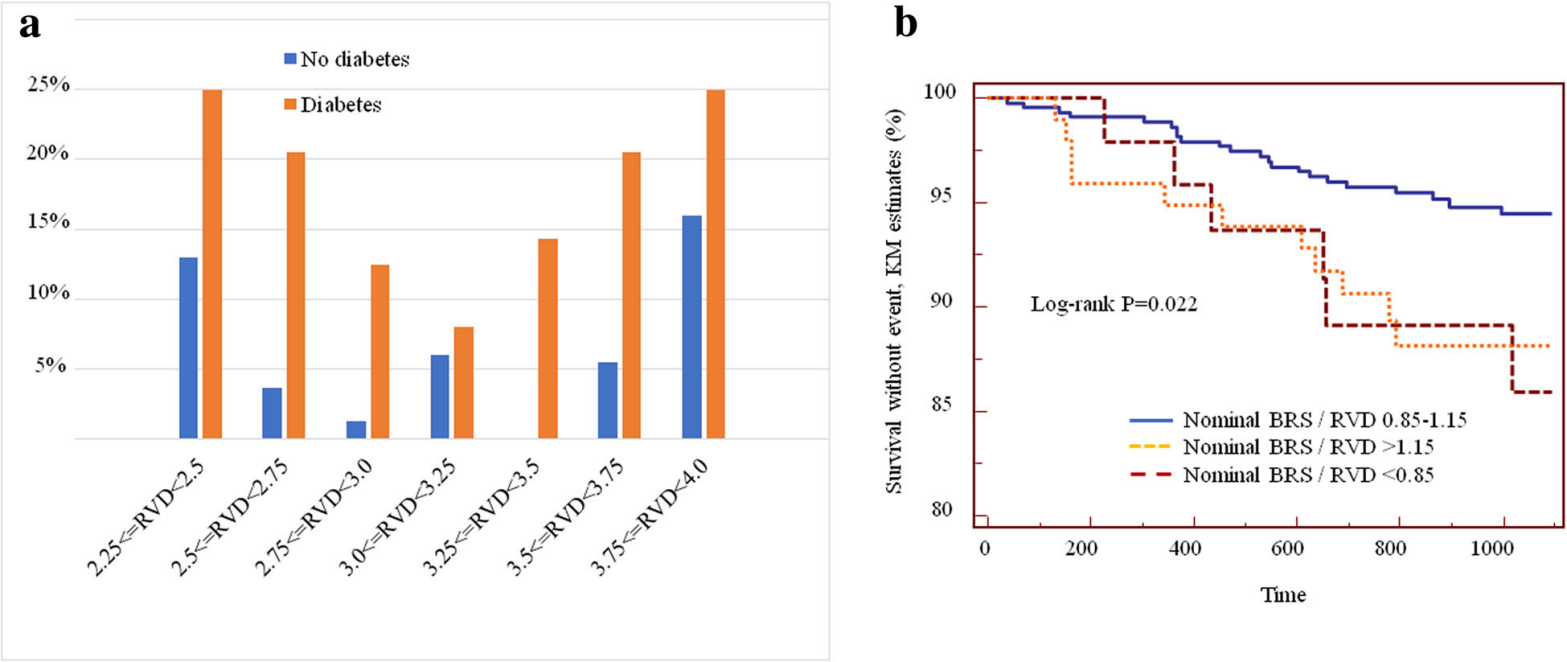
**a** Incidence of scaffold restenosis as a function of reference vessel diameter (RVD). The incidence of ScR described a U-shaped curve, with higher risk at both ends of the RVD spectrum. **b** Both oversizing (as defined by a BRS/RVD ratio > 1.15) and undersizing (BRS/RVD ratio < 0.85) were associated with increased ScR risk in diabetic patients

The incidence of ScR was higher in diabetics for all size of the target vessel (Fig. 3a). The relationship between incidence of ScR and RVD described a U-shaped curve, demonstrating an increasing risk at both ends of the RVD spectrum. Due to the lower incidence of ScR, this trend was less manifest in non-diabetics. When the risk of ScR was plotted against the relationship between nominal BRS diameter and RVD, an increased risk was shown for both undersized BRS (as defined by a BRS at least 15% smaller than the RVD, ie BRS/RVD ratio < 0.85) and oversized BRS (BRS/RVD ratio > 1.15) (Fig. 3b). Finally, in Cox-regression, RVD was a predictor of ScR in non-diabetics HR:0.38 [0.16–0.93], *P* = 0.035), but not in diabetics (HR: 0.46 [0.16–1.31], *P* = 0.148).

## Discussion

Diabetes is a known risk factor and determinant of prognosis after metallic stent implantation. Long-term evidence on the outcome of patients with diabetes treated with BRS is still scarce. Except for one study [15], data available report the incidence of events only during the first year after BRS implantation [12–14], a time that does not allow conclusions on the risk of ScR. We report on the impact of diabetes in a cohort of consecutive patients with a three-years follow-up. Similar to what observed by Muramatsu et al. [14], there was no significant difference between diabetes and non-diabetes patients in any of the outcomes during the first year after BRS implantation. After one year, however, diabetes was a predictor of cardiovascular death, target vessel- and target lesion-revascularization, and ScR. As a possible mechanistic explanation for this observation, the postprocedural residual stenosis, a procedural parameter that is known to be associated with worse outcomes [20], was significantly larger in the diabetes group. As previously published [21], the incidence of ScR appeared to be inversely proportional to the size of the target vessel, but the reference vessel diameter was a stronger predictor of ScR in non-diabetics as compared to diabetic patients. In the recent 1-year GHOST-EU analysis [13], a significant interaction between diabetes and reference vessel diameter ≤ 2.75 mm was shown, and in patients with larger vessels the impact of diabetes was mitigated (rate of 1-year device oriented composite event in patients with reference diameter > 2.75 mm: 5.7% in diabetics vs. 3.9%). Taken together, these findings may suggest that vessel size influences the timing (higher risk of device failure in patients with RVD < 2.75 mm during early follow-up), but not the long-term impact of diabetes (higher incidence of ScR in patients with diabetes independently of the vessel size). The present findings further expand these concepts. Taken as a continuous variable RVD was not a predictor of events. However, in patients with diabetes, the risk of ScR was progressively higher for both smaller and larger RVDs, describing a U-shaped curve with lowest incidence for RVDs between 2.75 and 3.5 mm. This evidence demonstrates that sizing is indeed important [10, 22]. In analogy, the incidence of ScR was higher in the presence of mismatch between nominal BRS size and vessel size (RVD). Of note, this finding is in line with computational fluid dynamics analyses showing that both stent/scaffold oversizing and undersizing cause blood flow disturbances and recirculation microenvironments both proximal and distal to the treated segment, resulting in a stimulus for neointima formation [23].

Importantly, the association between diabetes and ScR-related outcomes was maintained when differences in the cardiovascular risk profile were accounted for in a propensity score analysis. Finally, in diabetic patients in whom BRS had been implanted following a set of recommendations including BRS sizing, correct vessel size selection, accurate pre- and postdilation, the incidence of ScR was significantly reduced, demonstrating an impact of the technique used at the time of implantation also on long-term outcomes.

### Limitations

The retrospective, observational nature of the study does not allow inferring mechanistic conclusions. As well, despite accurate patient phenotypization with accepted clinical criteria, bias might exist which were not equalized with the propensity score. Although there was no external monitoring, 100% of the data were monitored by staff not responsible for data entry. As well, all events were adjudicated by experienced cardiologists based on original documents. Importantly, the classification into groups was based on the presence of diabetes at the time of treatment, and we cannot exclude that some of the patients in the control group had a new diagnosis of diabetes during follow-up. Levels of glycol-hemoglobin were not available, and (like in the previous papers [11–13, 15]) information on the impact of changes in medical or insulin treatment during follow-up was not collected. Of note, in the GHOST-EU subanalysis, diabetic patients treated with and without insulin had quite similar clinical, angiographic and procedural characteristics and nonsignificant differences outcomes [13]. The rate of ScT reported here is slightly higher than that reported by Muramatsu et al. [14], an observation that is likely associated with the higher risk profile and the inclusion of complex lesion in the present cohort, but it is lower than that described by Kereiakes et al. [12]. The incidence of cardiovascular death, but not that of diagnosed myocardial infarction, was higher in the diabetes group after 365 days. Although no documentation suggesting myocardial infarction or scaffold-related events was available in these cases, a very late event cannot in principle be excluded. Finally, although we show a difference in the outcomes in association with appropriate implantation technique, we cannot provide a direct comparison between BRS and newer generation drug eluting stents as recently performed in the UNDERDOGS study for long lesions [24]. For comparison, however, our reported incidence of no scaffold restenosis and 1.2% scaffold thrombosis at two years in the group of diabetics with appropriate implantation technique are in the same range of (if not lower than) those previously reported in patients treated with everolimus-eluting metallic stents [25].

## Conclusions

We provide long-term outcome data in patients treated with BRS. In line with previous evidence with metallic stents, one year after implantation, diabetes was associated with an increased incidence of ScR and related outcomes, a phenomenon that was more pronounced in vessels outside of the 2.75–3.5 mm range and in the presence of a mismatch between BRS and vessel size. Of note, this negative impact of diabetes was reset when an optimal implantation technique was used. Data on the outcome of these lesions after BRS resorption is completed are awaited.

## Additional file

Additional file 1: **Table S1.** A and B Patient characteristics after ATE adjustment (patient level). (DOCX 18 kb)

## Abbreviations

ATE: Average treatment effect
BRS: Bioresorbable drug-eluting vascular scaffold
CABG: Boronary artery bypass graft
CI: Confidence interval
CTO: Chronic total occlusion
CV: Cardiovascular
DES: Drug-eluting stent
DoCE: Device-oriented composite endpoint
eGFR: Estimated glomerular filtration rate
HR: Hazard ratio
IQR: Interquartile range
LAD: Left anterior descending
LCX: Left circumflex artery
LM: Left main stem
LVEF: Left ventricular ejection fraction
MI: Myocardial infarction
MICAT: Mainz Intracoronary Database. The Coronary Slow-flow and Microvascular Diseases Registery
MLD: Minimal lumen diameter
NSTEMI: Non-ST-elevation myocardial infarction
PCI: Percutaneous coronary intervention
QCA: Quantitative coronary analysis
RCA: Right coronary artery
RVD: Reference vessel diameter
ScR: Scaffold restenosis
ScT: Scaffold thrombosis
SD: Standard deviation
STEMI: ST-elevation myocardial infarction
TIA: Transitory ischemic attack
TLR: Target lesion revascularization
TVMI: Target vessel myocardial infarction
TVR: Target vessel revascularization
