# Supplementary material for: Three-years outcomes of diabetic patients treated with coronary bioresorbable scaffolds
Source: BMC Cardiovasc Disord. 2018 May 10;18:92. doi: 10.1186/s12872-018-0811-7 (PMC5944115; doi:10.1186/s12872-018-0811-7)
Supplement: Supplementary file 1 — Table S1. A and B Patient characteristics after ATE adjustment (patient level). (DOCX 18 kb) [file 12872_2018_811_MOESM1_ESM.docx]

Table S1a Patient characteristics after ATE adjustment (lesion level)

| Variable | All (1316.34) | no diabetes (658.68) | diabetes (657.66) | p |
| --- | --- | --- | --- | --- |
| Age (yrs) | 63.3 (12.1) | 63.1 (12.4) | 63.6 (11.8) | 0.66 |
| Male | 80.0% (1053/1316) | 80.2% (529/659) | 79.8% (525/658) | 0.89 |
| Hypertension | 73.8% (972/1316) | 72.2% (476/659) | 75.5% (496/658) | 0.34 |
| Smoking | 40.8% (538/1316) | 41.1% (271/659) | 40.6% (267/658) | 0.90 |
| Family History | 21.5% (283/1316) | 21.8% (144/659) | 21.2% (139/658) | 0.85 |
| Hyperlipidemia | 40.5% (533/1316) | 40.0% (263/659) | 41.0% (269/658) | 0.79 |
| Prior CABG | 1.8% (23/1316) | 1.6% (11/659) | 1.9% (13/658) | 0.77 |
| Prior PCI | 28.7% (377/1316) | 27.6% (181/659) | 29.8% (196/658) | 0.52 |
| Prior stroke/TIA | 4.4% (58/1316) | 4.2% (28/659) | 4.6% (30/658) | 0.80 |
| eGFR (ml/min) | 83.15 (23.18) | 82.81 (22.09) | 83.49 (24.29) | 0.77 |
| Silent/Stable angina | 32.6% (429/1316) | 33.1% (218/659) | 32.1% (211/658) | 0.79 |
| Unstable angina | 11.5% (152/1316) | 11.6% (76/659) | 11.5% (76/658) | 0.99 |
| NSTEMI | 29.8% (392/1316) | 30.1% (198/659) | 29.4% (194/658) | 0.86 |
| STEMI | 25.3% (333/1316) | 24.5% (161/659) | 26.1% (172/658) | 0.63 |

Table S1b Patient characteristics after ATE adjustment (patient level)

| Variable | All (1121.09) | no diabetes (563.93) | diabetes (557.15) | p |
| --- | --- | --- | --- | --- |
| Age (yrs) | 63.2 (12.2) | 63.0 (12.3) | 63.5 (12.1) | 0.73 |
| Male | 80.0% (897/1121) | 79.8% (450/564) | 80.3% (447/557) | 0.90 |
| Hypertension | 72.3% (810/1121) | 71.2% (401/564) | 73.4% (409/557) | 0.55 |
| Smoking | 42.5% (477/1121) | 42.7% (241/564) | 42.3% (236/557) | 0.92 |
| Family History | 20.2% (227/1121) | 21.0% (118/564) | 19.5% (109/557) | 0.66 |
| Hyperlipidemia | 39.7% (445/1121) | 39.4% (222/564) | 40.1% (223/557) | 0.86 |
| Prior CABG | 2.0% (22/1121) | 1.9% (11/564) | 2.1% (12/557) | 0.87 |
| Prior PCI | 27.3% (306/1121) | 26.4% (149/564) | 28.2% (157/557) | 0.65 |
| Prior stroke/TIA | 4.5% (50/1121) | 4.2% (24/564) | 4.7% (26/557) | 0.79 |
| eGFR (ml/min) | 83.38 (23.71) | 83.06 (22.25) | 83.71 (25.18) | 0.80 |
| Silent/Stable angina | 29.3% (328/1121) | 29.5% (166/564) | 29.1% (162/557) | 0.92 |
| Unstable angina | 12.1% (136/1121) | 11.9% (67/564) | 12.3% (69/557) | 0.86 |
| NSTEMI | 30.9% (347/1121) | 31.7% (179/564) | 30.1% (168/557) | 0.70 |
| STEMI | 27.5% (309/1121) | 26.7% (150/564) | 28.4% (158/557) | 0.64 |
